# miRNAs from Plasma Extracellular Vesicles Are Signatory Noninvasive Prognostic Biomarkers against Atherosclerosis in LDLr^−/−^Mice

**DOI:** 10.1155/2022/6887192

**Published:** 2022-08-17

**Authors:** Ke-feng Zhai, Hong Duan, Yan Shi, Ya-ru Zhou, Yuan Chen, Yao-shuai Zhang, Zi-peng Gong, Wen-gen Cao, Jia Wu, Jun-jun Wang

**Affiliations:** ^1^Department of Clinical Laboratory, Jinling Hospital, Medical School of Nanjing University, Nanjing 210002, China; ^2^Suzhou Engineering and Technological Research Center of Natural Medicine and Functional Food, School of Biological and Food Engineering, Suzhou University, 49, Bianhe Road, Suzhou 234000, China; ^3^State Key Laboratory for Chemistry and Molecular Engineering of Medicinal Resources (Guangxi Normal University), Guilin 541004, China; ^4^State Key Laboratory of Functions and Applications of Medicinal Plants, Guizhou Provincial Key Laboratory of Pharmaceutics, Engineering Research Center for the Development and Application of Ethnic Medicine and TCM (Ministry of Education), Guizhou Medical University, Guiyang 550004, China; ^5^Faculty of Pharmacy, Bengbu Medical College, Bengbu 233030, China

## Abstract

Circular microRNAs (miRNAs) have become central in pathophysiological conditions of atherosclerosis (AS). However, the biomarkers for diagnosis and therapeutics against AS are still unclear. The atherosclerosis models in low-density lipoprotein receptor deficiency (LDLr^−/−^) mice were established with a high-fat diet (HFD). The extraction kit isolated extracellular vesicles from plasma. Total RNAs were extracted from LDLr^−/−^ mice in plasma extracellular vesicles. Significantly varying miRNAs were detected by employing Illumina HiSeq 2000 deep sequencing technology. Target gene predictions of miRNAs were employed by related software that include RNAhybrid, TargetScan, miRanda, and PITA. Gene Ontology (GO) and Kyoto Encyclopedia of Genes and Genomes (KEGG) further analyzed the intersection points of predicted results. The results showed that the HFD group gradually formed atherosclerotic plaques in thoracic aorta compared with the control group. Out of 17, 8 upregulated and 9 downregulated miRNAs with a significant difference were found in the plasma extracellular vesicles that were further cross-examined by sequencing and bioinformatics analysis. Focal adhesion and Ras signaling pathway were found to be the most closely related pathways through GO and KEGG pathway analyses. The 8 most differentially expressed up- and downregulated miRNAs were further ascertained by TaqMan-based qRT-PCR. TaqMan-based qRT-PCR and in situ hybridization further validated the most differentially expressed miRNAs (miR-378d, miR-181b-5p, miR-146a-5p, miR-421-3p, miR-350-3p, and miR-184-3p) that were consistent with deep sequencing analysis suggesting a promising potential of utility to serve as diagnostic biomarkers against AS. The study gives a comprehensive profile of circular miRNAs in atherosclerosis and may pave the way for identifying biomarkers and novel targets for atherosclerosis.

## 1. Introduction

In the early nineties, microRNAs (miRNAs) were first discovered in Caen Elegans. The miRNA is a single-strand, endogenous, nonprotein-coding small RNA molecule of about 22 nucleotides [1]. The miRNAs have particular roles in gene regulation at the posttranscriptional level [2, 3]. The miRNA can discern the corresponding region of specific target mRNA and then interact with it, leading to mRNA degradation or expression suppression [4]. To date, almost 721 human and 579 mouse miRNAs have been affirmed, which regulate about 30% of protein-coding genes in human beings. One miRNA can control no less than one target gene, and multiple miRNAs can regulate the target genes. The modulations in miRNAs are associated with the development and progression of several diseases, such as atherosclerosis (AS), nasopharyngeal carcinoma (NPC), obesity, and diabetes [5–7]. Therefore, the miRNAs might be used as biomarkers in these diseases.

The most prevailing cause of coronary heart disease, cerebral infarction, and peripheral vascular disease is atherosclerosis, where the obstacle of lipid metabolism is the basis of atherosclerosis [8]. This chronic inflammatory disease is caused by the aggression of WBCs to the oxidized low-density lipoproteins (LDL) with the recruitment of activated endothelial cells (ECs) [9]. Atherosclerosis is characterized by increased oxidative stress associated with endothelial dysfunction, infiltration of leukocytes, and deposition of modified lipoproteins [10]. The harmful consequence of oxidized LDL further accumulates the cytokine secretions, growth factors, and leukocytes that collectively accelerate the lesion and inflammation that ultimately end up to atherosclerosis [5].

Presently, major clinical diagnostic methods of atherosclerosis include biochemical examination, blood profiling with a particular focus on lipids, and X-ray examination; however, there are still many niches in early and accurate diagnosis of atherosclerosis. The miRNA-based gene regulations are already in practice for diagnosing many diseases [11, 12]. Our present study was designed to detect miRNAs from extracellular vesicles (EVs) in plasma by high-throughput RNA sequencing that may serve as targets for further exploration on miRNA-based diagnostic tool against AS.

## 2. Material and Methods

### 2.1. Animals and Atherosclerosis Model

Low-density lipoprotein receptor (LDLr) deficiency male C57BL/6 mice (8 weeks old, 18 ± 2 g body weight) were obtained from Model Animal Research Center, Nanjing, China. The animals were kept at standard temperature 22 ± 2°C, with air conditioning and relative humidity of 55%-65% with 12 h light/12 h dark circulation. LDLr^−/−^ mice were divided into the control and high-fat diet (HFD) groups, with eight mice in each group. The mice in the HFD group were induced with atherosclerosis through a 12-week high-fat diet (LDLr^−/−^ mice were fed a high-fat diet containing 21% fat and 0.21% cholesterol (D12079B, Open Source Diets, Research Diets, Inc.)) [13–15]; in contrast, the mice in the control group were given a normal feed (LDLr^−/−^ mice were fed a standardized chow diet). The drinking water of the mice was disinfected by ultraviolet light, and the water bottle and the pad material were changed every other day. All animal handling processes complied with the application of the guidelines for experimental animal care of Suzhou University (SZU-19002).

### 2.2. Hematoxylin and Eosin (HE) Staining

The mice were anesthetized with pentobarbital sodium and fixed on the dissecting table to expose the chest cavity. The base of the ascending aorta and the brachial artery were separated after perfusion of the left ventricle with cold PBS and cold 4% paraformaldehyde. The aortic root was cut into 5 *μ*m sections, and paraffin was removed after paraffin embedding. HE staining was carried out finally after rehydration.

### 2.3. Isolation and Detection of Extracellular Vesicles

All mice were sacrificed and blood from both groups' mice were collected, centrifuged, and preserved for further analyses as described in earlier studies [16]. Blood samples were collected in EDTA blood tubes and centrifuged to separate the plasma fraction. Plasma samples were then stored at −80°C until further processing.

The exoEasy Maxi kit (Qiagen, 76064) was used to extract extracellular vesicles (EVs) from plasma according to manufacturer instructions [17]. The extracellular vesicle morphology was determined by a transmission electron microscope (TEM; JEM-1200EX, Japan) with a protocol modification as described by Rodriguez-Caro and Dragovic [18]. Briefly, 10 *μ*L of EVs was loaded onto Cu grids and incubated for 10 min at room temperature. They were then stained with 2% uranyl acetate (aqueous) for 2 min before air drying and examination by TEM. The sizes of the EVs were analyzed by nanoparticle tracking analysis (NTA) using a Zetaview (Particle Metrix) with a 488 nm laser [17]. All EV samples were diluted in PBS for 100 times before NTA and then analyzed according to manufacturer instructions. In addition, the presence of specific EV markers was determined by western blot. Briefly, isolated EVs were nurtured with lysis buffer. After resolving the protein samples (30 mg) on SDS-polyacrylamide gelelectrophoresis (SDS-PAGE), they were shifted to polyvinylidene difloride (PVDF) films. The loaded membranes were blocked with 5% skimmed milk and incubated with primary antibodies (Cell Signaling Technology, Beverly, MA, USA) and secondary antibodies (Bioworld Technology Inc, St. Louis Park, MN, USA) for overnight at 4°C and 2 h at room temperature, respectively. Immunoreactive protein bands and their intensities were visualized under the FluorChem HD2 Imaging system (Protein Simple, CA, USA) and Alpha View SA, respectively [19].

### 2.4. RNA Extraction

Total extracellular vesicle RNAs were extracted by Express miRNA Extraction Kit (Tissue and cell). After thawing the plasma, it was stored on ice for later use. The plasma was centrifuged at 2000 g and 4°C for 20 min for 20 min. The clear supernatant was taken to the new EP tube and then centrifuged at 10000 g and 4°C for 20 min, and again, a clear supernatant was shifted to the new EP tube. It was then added with 75 *μ*L PBS and mixed evenly, afterwards, and 7.5 *μ*L proteinase K was mixed with the sample and incubated for 10 min at 37°C. Then, it was added with 45 *μ*L outside secrete body sedimentation reagent (total extracellular vesicles isolation (from plasma)) and mixed at 4°C after incubation for 30 min. After the incubation of 30 min, the mixed solution was centrifuged at 10000 g and 4°C for 30 min. After centrifugation for 30 min, 50 *μ*L PBS was added to the supernatant and resuspended. Out of this prepared solution of microRNA, 300 *μ*L microRNA and Reagent A were mixed and allowed to settle for 5 min at room temperature and then mixed with Reagent B. The solution was centrifuged at 13000 g for 5 min at a low temperature (not higher than 4°C). Out of this solution, 550 *μ*L of the supernatant was shifted from this solution to a new EP tube containing 200 *μ*L of absolute ethanol. The mixture was left at room temperature for five minutes and then centrifuged under the same conditions for ten minutes. Then, moved 700 *μ*L to a new EP supernatant fluid tube and added 300 *μ*L isopropanol and blended. Two solutions in EP tubes were transferred to the adsorption column for centrifugation at 13000 g at 4°C for 1 min and abandoned the supernatant. Isopropanol (75%) for 700 *μ*L was added to the adsorption column, which then was centrifuged at 13000 *g* at 4°C for 1 min, and the supernatant was discarded. Adding 500 *μ*L of anhydrous ethanol to the adsorption column was centrifuged under the same conditions, and the supernatant was discarded. RNase-free TE buffer was added to the adsorption column filter and then allowed to settle for 2 min. After centrifugation, the product of extracted miRNAs was obtained by elution with eluent.

### 2.5. Screening Significant Differential miRNAs

Total RNA was extracted from the plasma of two groups of mice. HiSeq 2000 deep sequencing technology (Shenzhen BGI Co. Ltd., Wuhan, China) was applied to screen out the miRNAs with significant differences in expression [20, 21]. The false discovery rate (FDR) was indicated as the multiple difference between the control and model groups. The fold change (FC) and *p* values were calculated by applying the Student *t*-test [22].

### 2.6. Bioinformatics Analysis

Multiple bioinformatics software (TargetScan, miRanda, and RNAhybrid) were employed to predict differentially expressed miRNA targets [23]. After appropriate statistical analyses (formula) with a *p* value of less than 0.05, the common regions obtained from three software were further subjected to the KEGG and GO enrichment analyses [24].

Formula:

(1) $$\begin{array}{c} P=1-\overset{m-1}{\underset{i=0}{\sum }}\frac{\left(\begin{array}{c} M\\ i \end{array}\right)\left(\begin{array}{c} N-M\\ n-i \end{array}\right)}{\left(\begin{array}{c} N\\ n \end{array}\right)}. \end{array}$$

### 2.7. Confirmation of Expression of miRNAs

After a shortlisting of differentially expressed miRNAs through deep sequencing and bioinformatics software, the atherosclerosis-related miRNAs were further subjected to TaqMan-based qRT-PCR analyses to confirm diagnostic biomarkers as described earlier [25, 26]. The miRNAs were obtained from extracellular vesicles in plasma using Express miRNA Extraction Kit (Tissue and cell). All-in-One First-Strand cDNA Synthesis kit (Haigene Biotech, Haerbin, China) was used for reverse transcription for total RNAs using U6 snRNA as an internal reference gene [27, 28]. All samples were normalized to internal control, and fold changes were calculated through relative quantification.

### 2.8. In Situ Hybridization

In situ hybridization was performed as described previously [29]. The sections (5 *μ*m thick) were subjected to in situ hybridization kit (Exiqon, BOSTER Biological Technology Co. Ltd., China) following the manufacturer's instructions. DAPI was used as a nuclear counterstain. Slides were visualized using a confocal microscope (Olympus, Tokyo, Japan).

## 3. Results

### 3.1. Atherosclerosis Formation in LDLr^−/−^ Mice

**Figure 1:**
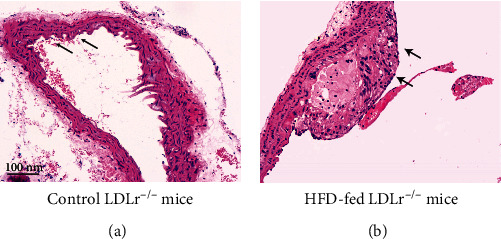
Assessment of the atherosclerotic status in LDLr^−/−^ mice. (a) Control group. (b) High-fat diet- (HFD-) fed group. Representative HE stains of aortic coronal vessel section (×200), scale bar 100 nm, *n* = 8.

To study the formation of atherogenesis *in vivo*, the plaque area and plaque composition in aortic roots were assessed by histopathological analyses. As shown in Figure 1, the LDLr^−/−^ mice treated with a high-fat diet (model group) displayed the most favorable plaque phenotype. The plaque and lesions were significantly larger than the control group, indicating that atherosclerosis developed successfully.

### 3.2. Extracellular Vesicle Detection

**Figure 2:**
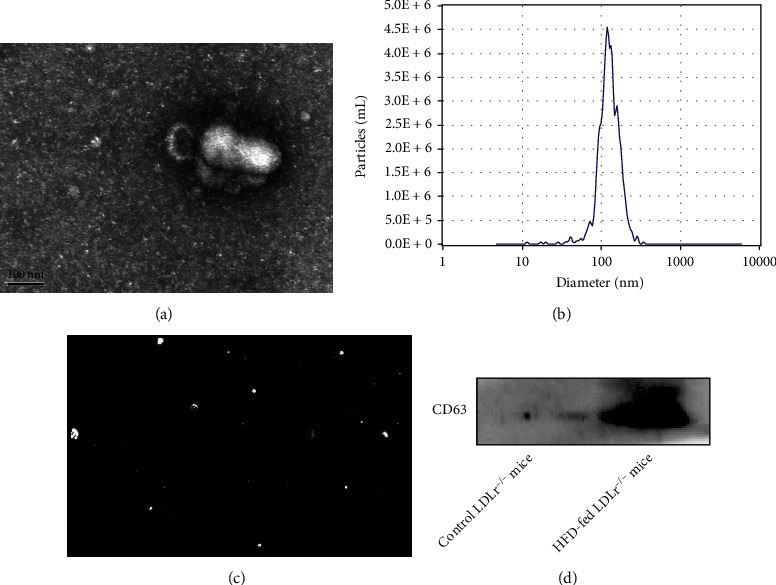
Detection of extracellular vesicles. (a) Representative TEM image of extracellular vesicles (scale bar 100 nm), *n* = 8. (b) Nanoparticle tracking analysis (NTA) of extracellular vesicles. (c) Video capture of recorded extracellular vesicle movements. (d) Standard markers CD63 was detected by western blot.

TEM analysis revealed that the extracellular vesicles from the plasma were irregular circular or elliptic structures with diameters of 30-200 nm. At the same time, the vesicles were observed to have a complete enveloped membrane (Figure 2(a)). Furthermore, we used NTA to determine the size and concentration of extracellular vesicles. As shown (Figures 2(b) and 2(c)), the diameters of plasma extracellular vesicles were 50 nm to 200 nm. The concentration of extracellular vesicles was 7.7 × 10^10^ particles/mL. Moreover, specific EV markers CD63 was perceived by western blot (Figure 1(d)). A large amount of CD63 was detected in the HFD-fed LDLr^−/−^ mice group in western blot experiment, indicating that a large amount of EVs was secreted in the HFD-fed LDLr^−/−^ mice group. The above experimental results showed that we had extracted and isolated good secretory EVs from plasma.

### 3.3. Classification of miRNAs

MicroRNA annotation statistics were identified by comparing them with the known sRNA database. To make each unique miRNA have a unique annotation, miRNAs were traversed and annotated according to the priority order of miRNA > pi-RNA > snoRNA > R-fam > others RNA [2, 30]. The information on mature and progeny miRNA was obtained by comparing the classification of annotation results and earlier reported database of mature miRNA. With the comparison of the miRNA database, some of the unknown (experimentally found) miRNAs were identified (Supplementary Table 1), while some other remaining unknown miRNAs were considered novel miRNAs. Thus, we have predicted some new miRNAs with the sequencing process (Supplementary Table 2).

### 3.4. Differentially Expressed miRNAs from Different Groups

Multiple software predicted the target genes of miRNAs with significant differences of expressions. In general, the miRNAs with a significant difference in an expression whose FDR was less than or equal to 0.001 and the multiple difference of it was more than two times were screened out. A total of 17 miRNAs showed a significant difference in expressions. Nine downregulated miRNAs (miR-421-3p, miR-350-3p, miR-184-3p, miR-331-3p, miR-700-3p, miR-6538, novel_ miR10, novel_miR18, and novel_miR23), and 8 upregulated miRNAs (miR-378d, miR-181b-5p, miR-107-3p, miR-146a-5p, miR-122-5p, miR-8112, miR-9b-3p, and novel_miR20) were found and listed (Table 1).

**Figure 3:**
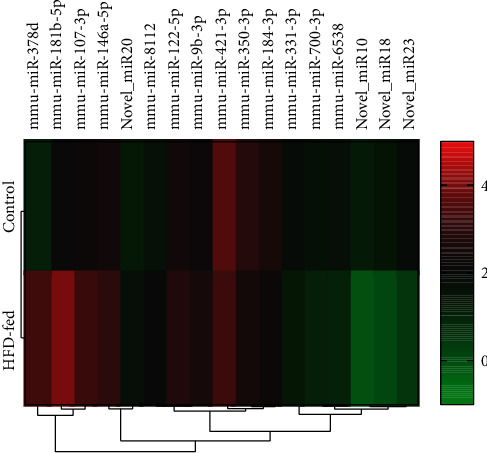
Heatmap of normalized miRNA reads that are differentially expressed between the control and HFD-fed groups, *n* = 8.

Heatmap function in R software was applied to hierarchical clustering analysis (Figure 3). Collectively, the normal mice and LDLr^−/−^ mice were correctly be separated because of 17 differentially expressed miRNAs by the clustering analysis (Figure 3). Therefore, these 17 miRNAs have the strong potential for their utility to detect atherosclerotic lesions.

### 3.5. Target Gene Prediction and Functional Analysis of miRNAs

**Figure 4:**
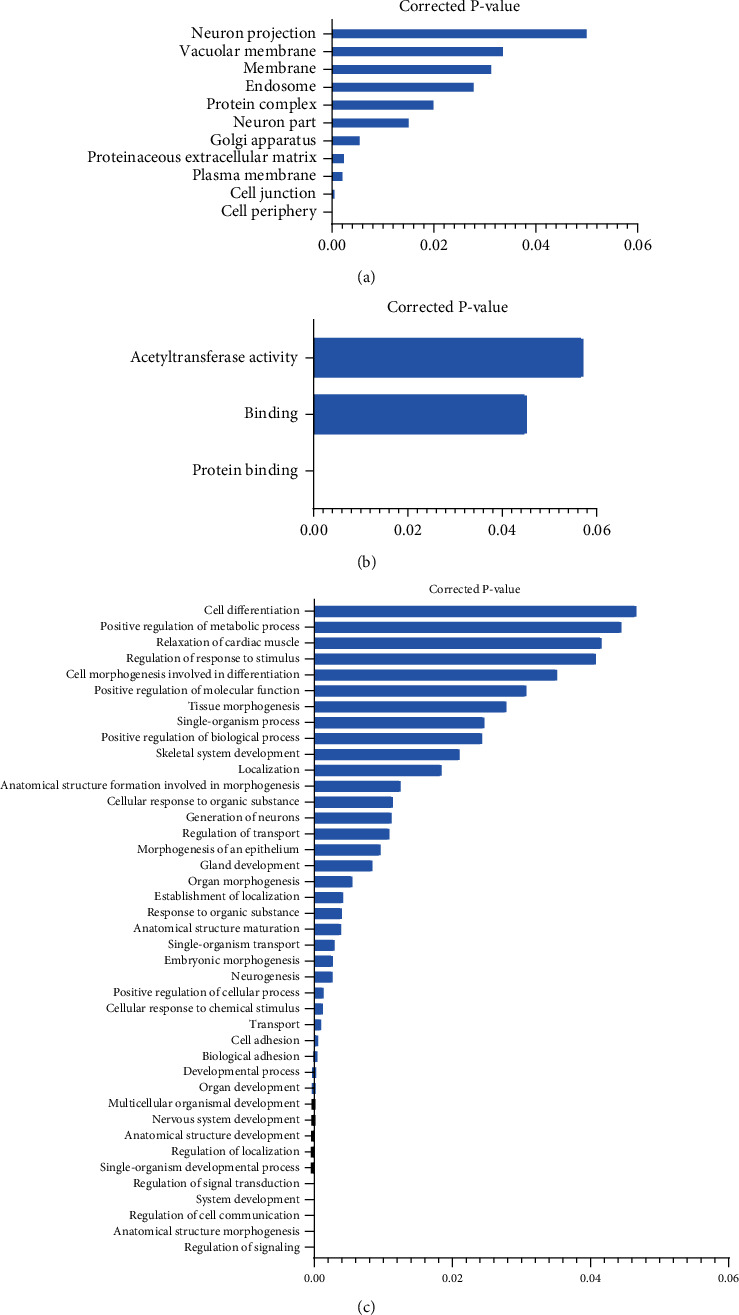
The *p* value of GO terms in experimental difference of the control vs. model. (a) GO analysis in cellular components. (b) GO analysis in molecular function. (c) GO analysis in biological processes (GO terms, which is significantly enriched in the target gene corresponding to differentially expressed sRNAs, is defined as *p* value ≤ 0.05. This figure only shows the GO term with *p* value ≤ 0.05).

Multiple bioinformatics software (TargetScan, PITA, miRanda, and RNAhybrid) were employed to predict differentially expressed miRNA target genes. The common intersecting regions from this four software indicated of 17 differentially expressed miRNAs (Supplementary Table 3). After appropriate statistical analyses and normalization for a *p* value of less than 0.05, the common regions obtained from these four software were further subjected to the GO significance enrichment analysis. The key functions incorporated for analysis included cellular components (CC), biological processes (BP), and molecular function (MF). GO function significance enrichment analysis was beneficial for determining the major biological functions of target genes of the differentially expressed sRNAs. These functioning entries in GO indicated the target genes' biological functions corresponding to the differentially expressed miRNA and their signal correlation. The results indicated that the differentially expressed genes were focused on cell periphery (Figure 4(a)), protein binding (Figure 4(b)), and regulation of signaling (Figure 4(c)), respectively.

**Figure 5:**
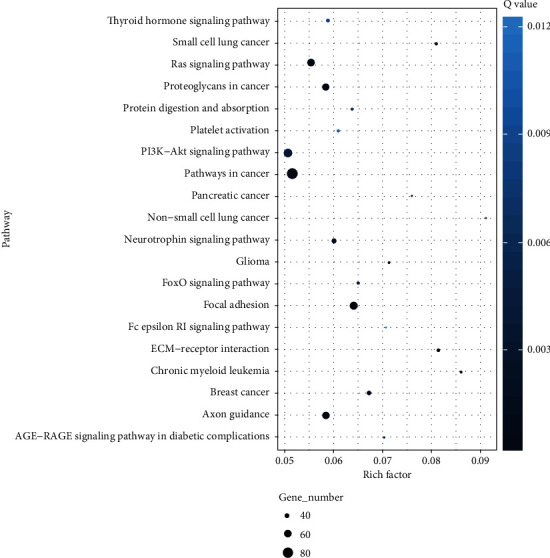
KEGG pathways of top 20 enrichment score.

**Figure 6:**
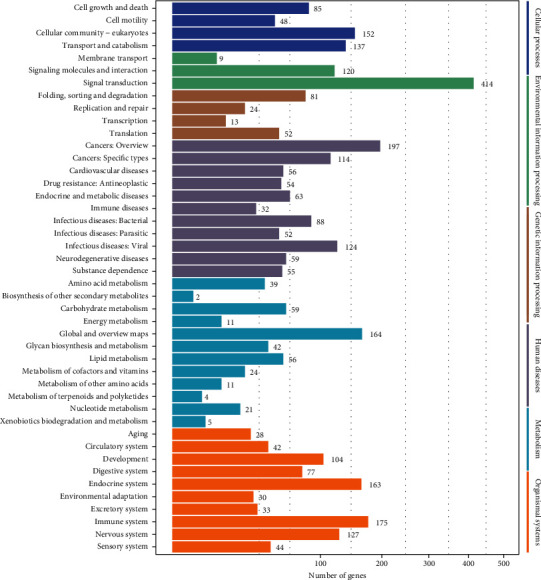
Classification statistics of KEGG channel annotation.

To further comprehend the biological functions and their corresponding pathways, KEGG was employed using single unit function. The hypergeometric test was assessed for the differentially expressed miRNAs target genes compared with the whole genome background. The top 20 pathways are shown in Figure 5. Focal adhesion and Ras signaling pathways were found to be strongly associated with AS. The KEGG classification counts the number and composition of proteins and genes (Figure 6). The results indicated cell progression and death; signal transduction against environmental factors; gene folding, sorting, and degradation; lipid metabolism; and circulatory and cardiovascular system may be closely connected with AS.

### 3.6. Validation of Expression of miRNAs

**Figure 7:**
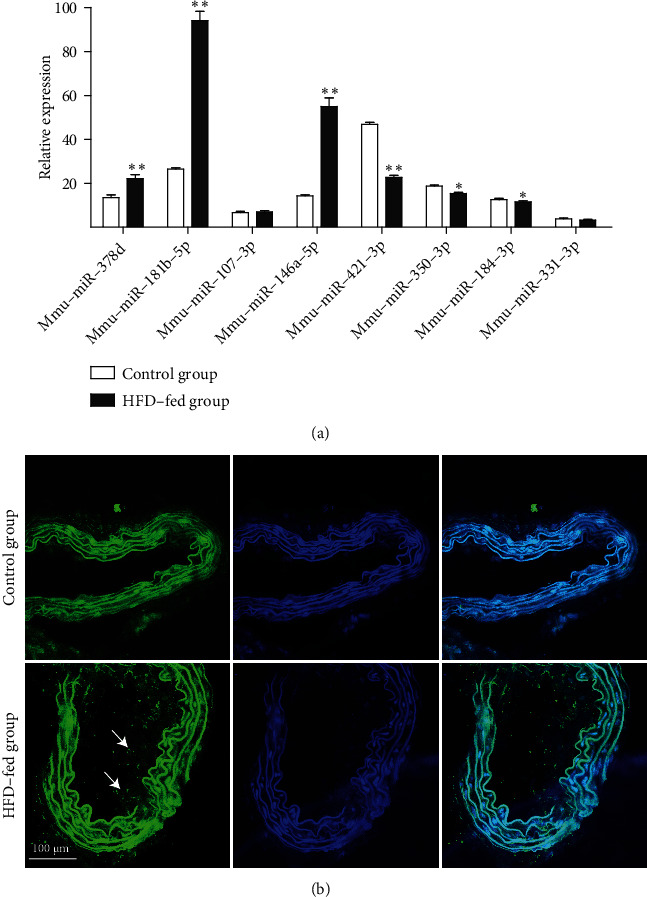
Validation of expression of miRNAs. (a) Validation by qRT-PCR. (b) The content of miR-146a in arterial tissue was evaluated by in situ hybridization from the mice. Scale bars 100 *μ*m. Each value represents the mean ± SEM (*n* = 6). ^∗^*p* < 0.05, ^∗∗^*p* < 0.01 vs. the control group.

As 17 differentially expressed miRNAs were attained from deep sequencing to obtain the most exact diagnostic biomarker of AS, the top 8 miRNAs (4 upregulated and 4 downregulated miRNAs) were assessed by TaqMan-based qRT-PCR. The relative levels of each miRNA were obtained and shown in Figure 7(a). mmu-miR-378d, mmu-miR-181b-5p, and mmu-miR-146a-5p in the AS group were observably increased compared to the control group (*p* < 0.05), respectively, while mmu-miR-107-3p showed no significant difference. mmu-miR-421-3p, mmu-miR-350-3p, and mmu-miR-184-3p in the AS group were markedly decreased compared with that in the control group (*p* < 0.05), while mmu-miR-331-3p showed no significant difference. In addition, in situ hybridization assay showed that miR-146a (green) in the HFD-fed group was significantly improved than in the control group (Figure 7(b)), indicating that the level of miR-146a is closely associated with the development of atherosclerosis. These results indicated that mmu-miR-378d, mmu-miR-181b-5p, mmu-miR-146a-5p, mmu-miR-421-3p, mmu-miR-350-3p, and mmu-miR-184-3p had good consistency with the deep sequencing results, which could serve as promising noninvasive biomarkers for AS.

## 4. Discussion

Atherogenesis was identified by the plaque area and plaque composition in aortic roots [31]. This is the first study to explore the effects of miRNA expression profiles in plasma extracellular vesicles of LDLr^−/−^ mice by combining two approaches, such as high-throughput sequencing and bioinformatics analyses. Our results suggested that the extracellular vesicle composition of plasma from mice with AS was far different than normal mice. The changes in plasma extracellular vesicle miRNA expression profiles may reflect the physiological and pathological processes of AS and may provide potential targets for treating AS [32].

In this study, 17 miRNAs were differentially expressed in extracellular vesicles extracted from the plasma of LDLr^−/−^ mice and normal mice. These included 8 upregulated miRNAs and 9 downregulated miRNAs that may be involved in developing AS. Out of these 17, 8 highly expressed miRNAs (4 upregulated and 4 downregulated miRNAs) were further assessed by TaqMan-based qRT-PCR. Our results showed the most differentially expressed miRNAs (miR-378d, miR-181b-5p, miR-146a-5p, miR-421-3p, miR-350-3p, and miR-184-3p) had good consistency with the deep sequencing results. Therefore, the six plasma miRNA panels mentioned above may have a promising diagnostic biomarker potential against AS.

The literature has confirmed that circulating miRNAs can be used as potential diagnostic biomarkers and prognostic factors in various diseases including AS [33–35]. Our data indicated that the upregulation of miR-378d, miR-181b-5p, and miR-146a-5p or downregulation of miR-421-3p, miR-350-3p, and miR-184-3p were closely related to AS. miR-378 and miR-421 are endogenous negative regulators of Ras signaling and cardiac hypertrophy and regulators of mitochondrial disruption and cardiomyocyte apoptosis, respectively [36–38]. Deleting miR-378 will lead to cardiac hypertrophy, and miR-421 may be a potential therapeutic target for heart disease. The miR-181 family is a target related to endothelial cell activation and immune cell homeostasis and plays an important role in vascular inflammatory responses by controlling key signaling pathways [37]. miR-146a mediates immune response and atherosclerotic inflammation, while miR-184 is involved in adipogenesis in PKP2-deficient cells [39, 40]. miR-350-3p is a new target for treating age-related inflammatory diseases due to its impaired age-related macrophage function [41]. In summary, atherosclerosis may be closely related to multiple biological processes that may directly or indirectly be involved in regulating the six miRNAs mentioned above.

EV miRNAs play an important role in diagnosing and treating multifactorial diseases, including T2DM and its cardiovascular complications. The collection of harvesting rare EV fractions or subpopulations increased the potential for miRNA biomarker discovery, and EV-shuttle miR-146a-5p improved its performance in detecting type 2 diabetes and its complications [42, 43]. Moreover, the inhibition of miR-1 reduced endothelial inflammation *in vitro* and attenuated atherogenesis in ApoE-deficient mice [44]. Endothelial microparticles promote vascular endothelial repair by delivering functional miR-126 into recipient cells [45]. miR-92a can be transported to macrophages through extracellular vesicles to regulate KLF4 levels, thus leading to the atheroprone phenotypes of macrophage and, hence, atherosclerotic lesion formation [46]. Endothelial microvesicle-mediated transfer of functional miR-92a-3p regulates angiogenesis in recipient endothelial cells by a THBS1- (thrombospondin 1-) dependent mechanism [47].

Reverse-transcription polymerase chain reaction (RT-PCR) is a powerful combination of assays useful in detection and measurement of expressed RNA transcripts. Currently, commonly used PCR-based procedures are standard, real-time, or quantitative (qPCR or qRT-PCR), or TaqMan [48]. We choose TaqMan-based qRT-PCR in this article. U6 belongs to small nuclear RNA (snRNA) in cells. U6 is stable, abundant, highly conserved, and ubiquitous in eukaryotic cells. So, we choose U6 snRNA as an internal reference gene [27, 28].

When we analyzed the potential function of differentially expressed miRNAs using the KEGG pathway, we found that the focal adhesion and Ras signaling pathway seemed to be strongly associated with the development of atherosclerosis [22, 49]. Adhesion of monocytes and lymphocytes to endothelium is also affected by other associated molecules inside endothelial cells [50], such as focal adhesion kinase (FAK). FAK is recruited to sites of focal adhesion (FA) and tightly linked to various structural networks of intracellular cytoskeletons and extracellular matrix (ECM) [40]. In addition, the Ras signaling pathway has been reported in the literature to participate in the cellular aging process [36]. Since Ras can mediate a variety of atherosclerotic stimuli including growth factors and oxidative stress, it is speculated that it can promote the aging of vascular cells and participate in the pathogenesis of atherosclerosis [36].

In summary, the differentially expressed genes (DEG) and related pathways that may be involved in AS in LDLr^−/−^ mice were comprehensively analyzed by bioinformatics in this study. We found a variety of miRNAs in plasma extracellular vesicles that are closely related to the physiological and pathological processes of AS and predicted the potential functions of related pathways and the targets of miRNAs. These results will facilitate the development of biomarkers as targets for diagnosing or treating AS.

## Figures and Tables

**Table 1:** Differentially expressed miRNAs from different groups (*n* = 8).

sRNA id	Count (control)	Count (model)	TPM (control)	TPM (model)	log2 ratio (model/control)	Up-downregulation (model/control)	*p* value	FDR
mmu-miR-378d	13	2165	1	168	7.3939481	Up	0	0
mmu-miR-181b-5p	103	10108	7.93	785	6.6297057	Up	0	0
mmu-miR-107-3p	199	1781	15.3	138	3.1749387	Up	0	0
mmu-miR-146a-5p	269	1039	20.7	80.7	1.9625986	Up	1.20*E* − 108	3.64*E* − 107
novel_mir20	19	61	1.46	4.74	1.6989187	Up	1.46*E* − 06	1.35*E* − 05
mmu-miR-8112	43	138	3.31	10.7	1.6954018	Up	3.46*E* − 13	4.16*E* − 12
mmu-miR-122-5p	271	658	20.9	51.1	1.2924572	Up	9.65*E* − 39	1.80*E* − 37
mmu-miR-9b-3p	156	346	12	26.9	1.162297	Up	4.14*E* − 18	5.50*E* − 17
mmu-miR-421-3p	4063	1918	313	149	-1.070159	Down	6.06*E* − 169	2.13*E* − 167
mmu-miR-350-3p	795	338	61.2	26.3	-1.2209	Down	3.14*E* − 42	6.27*E* − 41
mmu-miR-184-3p	412	174	31.7	13.5	-1.230298	Down	6.27*E* − 23	9.02*E* − 22
mmu-miR-331-3p	78	25	6.01	1.94	-1.631308	Down	1.34*E* − 07	1.31*E* − 06
mmu-miR-700-3p	48	13	3.7	1.01	-1.87317	Down	5.67*E* − 06	4.97*E* − 05
mmu-miR-6538	56	11	4.31	0.85	-2.342153	Down	1.57*E* − 08	1.62*E* − 07
novel_miR10	17	0	1.31	0	-10.35535	Down	8.26*E* − 06	7.04*E* − 05
novel_miR18	30	0	2.31	0	-11.17368	Down	1.07*E* − 09	1.14*E* − 08
novel_miR23	78	0	6.01	0	-12.55315	Down	4.70*E* − 24	7.11*E* − 23

## Data Availability

The data used to support the findings of this study are included within the article. The data used to support the findings of this study are included within the supplementary information file(s).

## Additional Points

*Key Points.* (i) The atherosclerosis model had been established in LDLr^−/−^ mice. (ii) Extracellular vesicles were isolated and identified in the plasma. (iii) Six plasma miRNA panels may serve as promising diagnostic biomarkers for AS. (iv) Circular miRNAs pave the way for biomarkers and novel targets for atherosclerosis.

## Supplementary Materials

Supplementary 1 Supplementary Table 1: the results of miRNA classification and annotation (*n* = 8).

Supplementary 2 Supplementary Table 2: unknown miRNA prediction results.

Supplementary 3 Supplementary Table 3: target gene prediction of 17 differentially expressed miRNAs by four software (TargetScan, PITA, miRanda, and RNAhybrid).
