# Supplementary material for: miRNAs from Plasma Extracellular Vesicles Are Signatory Noninvasive Prognostic Biomarkers against Atherosclerosis in LDLr−/−Mice
Source: Oxid Med Cell Longev. 2022 Aug 17;2022:6887192. doi: 10.1155/2022/6887192 (PMC9403256; doi:10.1155/2022/6887192)
Supplement: Supplementary 1 — Supplementary Table 1: the results of miRNA classification and annotation (n = 8). [file 6887192.f1.docx]

**Additional files:**

**Appendix 1: The results of** **miRNAs classification and annotation (n=8)**

| Type | Control group | | Model group | |
| --- | --- | --- | --- | --- |
|  | Count | Percentage (%) | Count | Percentage (%) |
| Total | 14051741 | 100 | 1.4E+07 | 100 |
| Intergenic | 44481 | 0.32 | 41692 | 0.31 |
| Mature | 10709904 | 76.22 | 1.1E+07 | 79.11 |
| R-fam other sncRNA | 3686 | 0.03 | 3421 | 0.03 |
| snRNA | 105 | 0 | 114 | 0 |
| Un-map | 1085818 | 7.73 | 705069 | 5.2 |
| Intron | 779525 | 5.55 | 662610 | 4.89 |
| Pi-RNA | 9409 | 0.07 | 14683 | 0.11 |
| rRNA | 57113 | 0.41 | 33786 | 0.25 |
| Hairpin | 18 | 0 | 17 | 0 |
| snoRNA | 10199 | 0.07 | 5430 | 0.04 |
| precursor | 525205 | 3.74 | 428601 | 3.16 |
| Exon | 690259 | 4.91 | 821962 | 6.06 |
| repeat | 134276 | 0.96 | 113455 | 0.84 |
| tRNA | 1743 | 0.01 | 2616 | 0.02 |
