# Supplementary material for: miRNAs from Plasma Extracellular Vesicles Are Signatory Noninvasive Prognostic Biomarkers against Atherosclerosis in LDLr−/−Mice
Source: Oxid Med Cell Longev. 2022 Aug 17;2022:6887192. doi: 10.1155/2022/6887192 (PMC9403256; doi:10.1155/2022/6887192)
Supplement: Supplementary 2 — Supplementary Table 2: unknown miRNA prediction results. [file 6887192.f2.doc]

**Additional files:**

**Appendix 2: Unknown miRNA prediction results**

| miRNA id | Chromosome | Strand | Sequence  (mature) | Sequence(star) | Start | End | Sequence  (precursor) | Tag info |
| --- | --- | --- | --- | --- | --- | --- | --- | --- |
| novel_mir1 | NC_000069.6 | - | uuuaguaguaccgucccuuu | ugguuaguacucugcguu | 96500345 | 96500422 | ugguuaguacucugcguuguggccgcagcaaccucgguucgaauccgagucacggcauuuaguaguaccgucccuuu | tag754_228,tag1826_231,tag4674_23,tag6135_31,tag2959_1154,tag5810_32,tag1780_66 |
| novel_mir2 | NC_000068.7 | - | agcuuguguugagccuguggau | ccagaggcucagcagaagcuua | 26020192 | 26020253 | agcuuguguugagccuguggauuuucucaauagcaagacccagaggcucagcagaagcuua | tag1556_15,tag5264_85 |
| novel_mir3 | NC_000067.6 | + | ugacugugccuguacauaugu | guaugugcauguacauacaug | 40259518 | 40259590 | ugacugugccuguacauaugugugugcaugugagcauaugugcauguacauguaugugcauguacauacaug | tag6350_37 |
| novel_mir3 | NC_000067.6 | + | ugacugugccuguacauaugu | guguguaugcacaucugu | 40259489 | 40259539 | guguguaugcacaucugugugugugcauaugacugugccuguacauaugu |  |
| novel_mir4 | NC_000068.7 | + | uagcacaaugugaaaagagcuc | gcccuuuuaacauugcacugcu | 84741117 | 84741178 | gcccuuuuaacauugcacugcucgguacacguuagacaguagcacaaugugaaaagagcuc | tag3637_80,tag3370_10,tag2371_14,tag3012_13 |
| novel_mir5 | NC_000073.6 | + | uggucuagggguaugauucu | uagcuucccuaguaagaacuggu | 98815373 | 98815441 | uagcuucccuaguaagaacuggugaagccugccuagcagcuggcucguuggucuagggguaugauucu | tag4269_29,tag3801_16,tag3564_141 |
| novel_mir6 | NC_000072.6 | - | auggagagacuuugacagug | uagccaagggaucuucaaau | 59705791 | 59705844 | uagccaagggaucuucaaaugcaugccagaguaauggagagacuuugacagug |  |
| novel_mir7 | NC_000075.6 | + | uagguagaccaggcugauc | uccuccugucucugccuucu | 122981876 | 122981935 | uagguagaccaggcugaucuagccacgaacucacagagauccuccugucucugccuucu | tag2652_11,tag2225_31 |
| novel_mir8 | NC_000073.6 | + | uagguagaccaggcugaua | auagucugcuuuugcuuucu | 113518140 | 113518193 | uagguagaccaggcugauauugaacucacaauuauagucugcuuuugcuuucu | tag4413_10 |
| novel_mir9 | NC_000077.6 | - | aacagaacugaaggacauacga | ucaugaccuucuggccugguuuu | 54012472 | 54012527 | ucaugaccuucuggccugguuuuuaucuauugaaacagaacugaaggacauacga | tag4465_83 |
| novel_mir10 | NC_000086.7 | + | uugguuuucggaacugaggcc | uggcagcugaaggcggcac | 24008723 | 24008796 | uugguuuucggaacugaggccauaauuaagaggaaggccaaggugguggugugguggcagcugaaggcggcac | tag4471_17 |
| novel_mir11 | NC_000080.6 | - | ugagguaguggguuguguu | ugcuugaugccggccgucucuagc | 65530947 | 65530994 | ugagguaguggguuguguuaauaugcuugaugccggccgucucuagc |  |
| novel_mir12 | NC_000075.6 | + | ugugggaaggaacuacaagaca | acuugugguuuuacuugacucacagg | 15313866 | 15313929 | acuugugguuuuacuugacucacaggacugacuguuaggucugugggaaggaacuacaagaca | tag5423_11,tag823_170,tag6315_24 |
| novel_mir13 | NC_000072.6 | - | auggagagacuuugacaggg | cugugggaucuucaaau | 60071103 | 60071153 | cugugggaucuucaaaugcaugccagaggaauggagagacuuugacaggg |  |
| novel_mir14 | NC_000068.7 | + | ucccuguccuccaggagcug | ccucaaguagacauggaga | 115656744 | 115656801 | ccucaaguagacauggagaaaauacaaaaacagaacgucccuguccuccaggagcug |  |
| novel_mir12 | NC_000075.6 | + | ugugggaaggaacuacaagaca | uuuuguuuguuuguuuuuaacuuuagg | 15313907 | 15313990 | ugugggaaggaacuacaagacagcuaacaaaguauaaaacugauguguuaauuuuguuuuguuuguuuguuuuuaacuuuagg |  |
| novel_mir15 | NC_000067.6 | - | cggggucuccccccgccgggcg | cucggcgggguccccgcg | 167340422 | 167340479 | cucggcgggguccccgcguccuccccgcagcggcgcggggucuccccccgccgggcg | tag2960_16,tag712_181,tag3192_21,tag1010_12 |
| novel_mir16 | NC_000081.6 | + | ucuuuugcuagaugcugugc | acgggugaucuagcagaagaug | 58935897 | 58935956 | ucuuuugcuagaugcugugccaaauccuggagauggcacgggugaucuagcagaagaug | tag4449_32 |
| novel_mir17 | NC_000077.6 | - | ugacugaaucuuguuaaagaau | ucuuuaacaagauucagucaca | 20706948 | 20707007 | ugacugaaucuuguuaaagaauagaaugaaaaaugcuucuuuaacaagauucagucaca | tag1995_28 |
| novel_mir18 | NC_000078.6 | + | caggaucuguaacuccagugu | acugggguuacagauccugca | 64973446 | 64973506 | caggaucuguaacuccagugucugccccauccacuucacacugggguuacagauccugca | tag5348_15,tag2565_15 |
| novel_mir15 | NC_000075.6 | - | cggggucuccccccgccgggcg | cuaggcaagguccccgcg | 105819342 | 105819399 | cuaggcaagguccccgcgucguccccgcggcggcgcggggucuccccccgccgggcg |  |
| novel_mir19 | NC_000068.7 | - | gguggcgacucuggacgcgagc | ugcggugggugucacagc | 73012854 | 73012921 | gguggcgacucuggacgcgagcugggcccuucccauggauggccucagcugcggugggugucacagc | tag748_10,tag4019_26,tag141_61 |
| novel_mir20 | NC_000070.6 | + | gaggaucugguucuguagcu | cuacaggaugagauccgguc | 41140171 | 41140230 | cuacaggaugagauccggucucaaaaaauuauuuuuggagaggaucugguucuguagcu | tag2406_80 |
| novel_mir21 | NC_000076.6 | + | cgagugacuugguaaauacga | guauuuaccaagucacucggu | 93688546 | 93688597 | guauuuaccaagucacucggucucuauaaccgagugacuugguaaauacga | tag2302_39,tag3489_11 |
| novel_mir22 | NC_000076.6 | - | cgagugacuugguaaauacg | uauuuaccaagucacucggu | 93688545 | 93688594 | uauuuaccaagucacucgguuauagagaccgagugacuugguaaauacg |  |
| novel_mir23 | NC_000082.6 | - | ccguggcgcaaugaaggu | acacacgccauggca | 57391606 | 57391692 | acacacgccauggcagacggaccaaggagucuaacgcgugcgcgagucaggggcucguccgaaagccgccguggcgcaaugaaggu | tag6104_10,tag3541_17,tag4769_12,tag1613_10,tag3456_13,tag5969_16 |
| novel_mir24 | NC_000076.6 | - | gauauguguguguaugucugugu | acagacauacacagacauacaga | 85886476 | 85886534 | gauauguguguguaugucuguguauauguauacacacagacauacacagacauacaga |  |
| novel_mir25 | NC_000074.6 | - | uuuagaaguguugggcuuu | agcccuccacugcucugaggg | 122270658 | 122270715 | agcccuccacugcucugaggggugccucgcaguuuaccuuuagaaguguugggcuuu | tag600_34 |
| novel_mir26 | NC_000081.6 | - | aggguuugagucaugacuuug | aauucaagauucaaaccuca | 83592226 | 83592288 | aggguuugagucaugacuuugcuaccuauauaagauguggcaaauucaagauucaaaccuca | tag4542_71,tag4727_30 |
