# Supplementary material for: miRNAs from Plasma Extracellular Vesicles Are Signatory Noninvasive Prognostic Biomarkers against Atherosclerosis in LDLr−/−Mice
Source: Oxid Med Cell Longev. 2022 Aug 17;2022:6887192. doi: 10.1155/2022/6887192 (PMC9403256; doi:10.1155/2022/6887192)
Supplement: Supplementary 3 — Supplementary Table 3: target gene prediction of 17 differentially expressed miRNAs by four software (TargetScan, PITA, miRanda, and RNAhybrid). [file 6887192.f3.pdf]

Additional files Table 2 (Appendix 2, Excel version) cannot be uploaded to the system. Now, Appendix 2 have been converted to PDF version and upload to system. Due to the large number of pages (608 pages), we only provide parts. After receiving the paper, we provide complete data (raw data) as supplementary data.

miRNA id	Target id	TargetScan type	TargetScan pct	TargetScan conserved	miRanda MFE	miRanda score	Symbol	GO term	Pathway	NR id	NR evalue	NR description
mmu-miR-378d	XM_006532949.2	7mer-m8	0	NA	-49	311	Git1	GO:0005829,GO:0016235,GO:0043005,GO:0045202,GO:0005925,GO:0016020,GO:0046872,GO:0032403,GO:0042803,GO:0005096,GO:0031267,GO:0032013,GO:0008277,GO:0032465,GO:0060996,GO:0001771,GO:0048013,GO:0043547,	K05737|1|0.0|1558|mmu:216963|G protein-coupled receptor kinase interactor 1	gi|568973161|ref|XP_006533012.1|	0	PREDICTED: ARF GTPase-activating protein GIT1 isoform X1 [Mus musculus]
mmu-miR-378d	XM_006496578.3	7mer-m8	0	NA	-71.7	470	Sphkap	-	K16527|1|8.11e-30|135|xtr:100145197|A-kinase anchor protein 11	gi|568907862|ref|XP_006496641.1|	0	PREDICTED: A-kinase anchor protein SPHKAP isoform X4 [Mus musculus]
mmu-miR-378d	XM_006513177.2	8mer-1a	0	NA	-27.6	161	Col6a2	-	K06238|1|0.0|1680|mmu:12834|collagen, type VI, alpha	gi|568966584|ref|XP_006513240.1|	0	PREDICTED: collagen alpha-2(VI) chain isoform X1 [Mus musculus]
mmu-miR-378d	NM_033652.5	8mer-1a	0	NA	-45.1	312	Lmx1a	GO:0005634,GO:0008270,GO:0000977,GO:0007411,GO:0045665,GO:0007626,GO:0007613,GO:0001558,GO:1904948,GO:0042048,GO:0090263,GO:0021549,GO:0050808,GO:0045944,GO:0021542,	K09371|1|0.0|798|mmu:110648|LIM homeobox transcription factor 1	gi|17298670|ref|NP_387501.1|	0	LIM homeobox transcription factor 1-alpha [Mus musculus]
mmu-miR-378d	NM_001163703.1	7mer-m8	0	NA	-91.6	633	Dcun1d3	GO:0000151,GO:0048471,GO:0005886,GO:0031624,GO:0032182,GO:0097602,GO:0010332,GO:0010225,GO:0051443,GO:0030308,GO:0043065,GO:0045116,	K17823|1|0.0|629|mmu:233805|DCN1-like protein 3	gi|27734156|ref|NP_775584.1|	0	DCN1-like protein 3 [Mus musculus]

mmu-miR-378d	NM_009898.3	6mer	0	NA	-26	165	Coro1a	GO:0001772,GO:0043234,GO:0030027,GO:0005884,GO:0045335,GO:0030864,GO:0001891,GO:0030424,GO:0070062,GO:0005911,GO:0005769,GO:0003785,GO:0042803,GO:0043548,GO:0008022,GO:0032036,GO:0051015,GO:0043524,GO:0032796,GO:0043320,GO:0050918,GO:0048873,GO:0001845,GO:0051279,GO:0030595,GO:0031339,GO:0051126,GO:0031589,GO:0030335,GO:0071353,GO:0061502,GO:0038180,GO:0008360,GO:0043029,GO:0042102,	K13882|1|0.0|950|mmu:12721|coronin-1A	gi|6753492|ref|NP_034028.1|	0	coronin-1A isoform 1 [Mus musculus]
mmu-miR-378d	NM_001039493.1	8mer-1a	0	NA	-50	320	Plekhm3	GO:0046872,	K19330|1|2.18e-43|174|ame:413668|run domain Beclin-1 interacting and cysteine-rich containing protein	gi|87162468|ref|NP_001034582.1|	0	pleckstrin homology domain-containing family M member 3 [Mus musculus]

mmu-miR-378d	XM_006528052.3	7mer-m8	0	NA	-32.3	180	Taf1	GO:0045120,GO:0005669,GO:0071339,GO:0002039,GO:0004402,GO:0004674,GO:0003713,GO:0017025,GO:0003677,GO:0070577,GO:0005524,GO:0006352,GO:0018107,GO:0046777,GO:0032436,GO:0018105,GO:0045944,GO:0016573,GO:0007049,	K03125|1|0.0|3917|mmu:270627|transcription initiation factor TFIID subunit 1	gi|134039180|sp|Q80UV9.2|TAF1_MOUSE	0	RecName: Full=Transcription initiation factor TFIID subunit 1; AltName: Full=Cell cycle gene 1 protein; AltName: Full=TBP-associated factor 250 kDa; Short=p250; AltName: Full=Transcription initiation factor TFIID 250 kDa subunit; Short=TAF(II)250; Short=TAFII-250; Short=TAFII250
mmu-miR-378d	NM_145551.4	7mer-1a	0	NA	-45.3	312	Slc5a9	-	K14389|1|0.0|1066|mmu:230612|solute carrier family 5 (sodium/glucose cotransporter), member 9	gi|568928906|ref|XP_006503064.1|	0	PREDICTED: sodium/glucose cotransporter 4 isoform X2 [Mus musculus]
mmu-miR-378d	NM_029098.3	6mer	0	NA	-41.2	301	Lmbr11	GO:0016021,GO:0005886,GO:0006897,	K00858|1|3.18e-16|87.8|smm:Smp_126160|NAD+ kinase [EC:2.7.1.23]	gi|755552260|ref|XP_011244060.1|	0	PREDICTED: protein LMBR1L isoform X1 [Mus musculus]
mmu-miR-378d	XM_011251006.2	8mer-1a	0	NA	-27.1	170	Tenm1	GO:0005856,GO:0005887,GO:0005576,GO:0005737,GO:0005634,GO:0046982,GO:0042803,GO:0006355,GO:0007218,	K06252|1|3.86e-52|207|xtr:100491528|tenascin	gi|569009290|ref|XP_006541555.1|	0	PREDICTED: teneurin-1 isoform X3 [Mus musculus]

mmu-miR-378d	NM_033587.3	8mer-1a	0	NA	-26.7	159	Pcdhga4	GO:0016021,GO:0005886,GO:0005509,GO:0007156,	K16495|1|0.0|1895|mmu:93712|protocadherin gamma subfamily A!K16497|3|0.0|1591|mcc:702071|protocadherin gamma subfamily C	gi|148747499|ref|NP_291065.3|	0	protocadherin gamma-A4 precursor [Mus musculus]
mmu-miR-378d	NM_011099.3	7mer-m8	0	NA	-44.4	298	Pkm	GO:0030955,GO:0004743,GO:0000287,GO:0016301,GO:0006096,	K00873|1|0.0|991|cge:100751347|pyruvate kinase [EC:2.7.1.40]	gi|568960835|ref|XP_006510919.1|	0	PREDICTED: pyruvate kinase PKM isoform X4 [Mus musculus]
mmu-miR-378d	NM_010800.4	8mer-1a	0	NA	-48	310	Bhlha15	GO:0005634,GO:0000977,GO:0001228,GO:0042803,GO:0048469,GO:0007186,GO:0007267,GO:0010832,GO:0007030,GO:0042149,GO:0006851,GO:0019722,GO:0045944,GO:0048312,GO:0030968,GO:0042593,	K08040|1|1.37e-141|396|mmu:17341|class B basic helix-loop-helix protein 8	gi|6754700|ref|NP_034930.1|	###	class A basic helix-loop-helix protein 15 [Mus musculus]
mmu-miR-378d	NM_153795.2	7mer-m8	0	NA	-41.4	296	Fermt3	GO:0030054,GO:0002102,GO:0070062,GO:0042995,GO:0016020,GO:0005178,GO:0070527,GO:0007229,GO:0033632,GO:0034446,GO:0030335,GO:0007159,GO:0033622,	K17084|1|0.0|1377|mmu:108101|kindlin 3	gi|24418903|ref|NP_722490.1|	0	fermitin family homolog 3 [Mus musculus]
mmu-miR-378d	NM_011066.3	7mer-1a	0	NA	-25.2	157	Per2	GO:0005654,GO:0005737,GO:0000989,GO:0005515,GO:0007623,GO:0000122,	K02633|1|0.0|2284|mmu:18627|period circadian protein	gi|568908375|ref|XP_006529313.1|	0	PREDICTED: period circadian protein homolog 2 isoform X2 [Mus musculus]
mmu-miR-378d	XM_006538872.3	6mer	0	NA	-43.2	297	Nphp4	GO:0035869,GO:0005813,GO:0097546,GO:0005923,GO:0032391,GO:0005737,GO:0097470,GO:0005515,GO:1903348,GO:0030317,GO:0090090,GO:0045494,GO:0060041,GO:0035845,	K16478|1|0.0|2909|mmu:260305|nephrocystin-4	gi|568931246|ref|XP_006538930.1|	0	PREDICTED: nephrocystin-4 isoform X2 [Mus musculus]

mmu-miR-378d	NM_010788.4	8mer-1a	0	NA	-26.8	172	Mecp2	GO:0000790,GO:0043234,GO:0005829,GO:0000792,GO:0005615,GO:0005739,GO:0098794,GO:0045322,GO:0047485,GO:0035197,GO:0008327,GO:0003729,GO:0008134,GO:0010385,GO:0003700,GO:0019904,GO:0000400,GO:0031490,GO:0042826,GO:0032048,GO:0043524,GO:0001976,GO:0006020,GO:0001662,GO:0048712,GO:0010288,GO:0046470,GO:0008284,GO:0001964,GO:0007507,GO:0051965,GO:0009405,GO:0042551,GO:0002087,GO:0031061,GO:0008211,GO:0016358,GO:0009790,GO:0008104,GO:0019233,GO:0006541,GO:0009791,GO:0006122,GO:0006349,GO:0006342,GO:0008542,GO:0001666,GO:0021591,GO:0035176,GO:0010212,GO:0008344,GO:0060079,GO:0000122,GO:0032355,	K11588|1|0.0|952|mmu:17257|methyl CpG binding protein 2	gi|14149645|ref|NP_034918.1|	0	methyl-CpG-binding protein 2 isoform 2 [Mus musculus]
mmu-miR-378d	NM_009931.2	8mer-1a	0	NA	-46.9	309	Col4a1	-	K06237|1|0.0|2973|mmu:12826|collagen, type IV, alpha	gi|161484654|ref|NP_034061.2|	0	collagen alpha-1(IV) chain precursor [Mus musculus]
mmu-miR-378d	NM_175331.3	7mer-1a	0	NA	-32.4	184	Nt5dc3	GO:0043235,GO:0005829,GO:0005739,GO:0052867,GO:0008253,GO:0046872,GO:0052830,GO:0052825,GO:1990003,GO:0043812,GO:0030352,GO:0030351,GO:0019178,GO:0008579,GO:0019198,GO:0052828,GO:0043726,GO:0052829,GO:0008330,GO:0017017,GO:0030487,GO:0017161,GO:0052629,GO:0052831,GO:0043813	K01081|1|5.96e-26|113|dpx:DAPPUDRAFT_216771|5'-nucleotidase [EC:3.1.3.5]	gi|568966237|ref|XP_006513080.1|	0	PREDICTED: 5'-nucleotidase domain-containing protein 3 isoform X1 [Mus musculus]

mmu-miR-378d	NM_028769.5	7mer-m8	0	NA	-45.7	294	Syvn1	GO:0036513,GO:0005654,GO:0005790,GO:0044322,GO:0000839,GO:0016021,GO:0070740,GO:0051087,GO:0070738,GO:0043773,GO:0008270,GO:1990381,GO:0043774,GO:0008766,GO:0070736,GO:0051117,GO:0051082,GO:0070737,	K10601|1|0.0|1234|mmu:74126|E3 ubiquitin-protein ligase synoviolin [EC:2.3.2.27]	gi|258547102|ref|NP_083045.4|	0	E3 ubiquitin-protein ligase synoviolin precursor [Mus musculus]
mmu-miR-378d	NM_013753.2	8mer-1a	0	NA	-45.1	308	Pald1	GO:0005829,GO:0005634,GO:0004725,	-	gi|568967679|ref|XP_006513768.1|	0	PREDICTED: paladin isoform X1 [Mus musculus]
mmu-miR-378d	NM_146122.3	8mer-1a	0	NA	-25.7	154	Denn d1a	-	K20160|1|0.0|1863|mmu:227801|DENN domain-containing protein 1	gi|568913605|ref|XP_006498063.1|	0	PREDICTED: DENN domain-containing protein 1A isoform X3 [Mus musculus]
mmu-miR-378d	NM_009971.1	7mer-m8	0	NA	-25.3	150	Csf3	GO:0005615,GO:0008083,GO:0005125,GO:0005130,GO:0019899,GO:0033138,GO:0030838,GO:0030851,GO:0008284,GO:0032092,GO:0006955,GO:0071345,GO:0042993,GO:2000251,GO:0014068,GO:1901215,GO:0045944,GO:0050731,GO:0051897,GO:0045639,	K05423|1|1.73e-145|407|mmu:12985|granulocyte colony-stimulating factor	gi|6753536|ref|NP_034101.1|	###	granulocyte colony-stimulating factor precursor [Mus musculus]
mmu-miR-378d	NM_001039472.1	7mer-m8	0	NA	-43.8	313	Kif21b	-	K10395|1|0.0|3363|mmu:16565|kinesin family member 4/21/27	gi|86990454|ref|NP_001034561.1|	0	kinesin-like protein KIF21B [Mus musculus]
mmu-miR-378d	XM_011243589.2	7mer-m8	0	NA	-42.1	310	Spec cl1	GO:0005815,GO:0005921,GO:0031941,GO:0005819,GO:0005737,GO:0007026,GO:0016477,GO:0007049,GO:0060325,GO:0051301,GO:0030835,GO:0007155,	K10388|1|2.33e-10|70.9|ecb:100147640|plectin	gi|224922835|ref|NP_700455.3|	0	cytospin-A isoform b [Mus musculus]

mmu-miR-378d	NM_177091.5	6mer	0	NA	-75.5	488	Fndc7	GO:0005576,	K16628|1|1.56e-14|83.6|hgl:101718816|collagen, type VII, alpha!K05717|5|5.19e-13|78.6|acs:100563512|fibronectin 1	gi|260166640|ref|NP_796065.2|	0	fibronectin type III domain-containing protein 7 isoform 1 precursor [Mus musculus]
mmu-miR-378d	NM_130454.2	7mer-m8	0	NA	-46.9	314	Recq15	GO:0005737,GO:0005694,GO:0016591,GO:0000993,GO:0043140,GO:0003677,GO:0005524,GO:0009378,GO:0032508,GO:1990414,GO:0051304,GO:2000042,GO:0051301,GO:0035690,GO:0034244,GO:0072757,GO:0006260,	K10902|1|0.0|2032|mmu:170472|ATP-dependent DNA helicase Q5 [EC:3.6.4.12]	gi|568971932|ref|XP_006532421.1|	0	PREDICTED: ATP-dependent DNA helicase Q5 isoform X1 [Mus musculus]
mmu-miR-378d	NM_025974.2	8mer-1a	0	NA	-41.1	298	Rpl14	GO:0005913,GO:0070062,GO:0016020,GO:0022625,GO:0098641,GO:0003735,GO:0006412,GO:0042273,GO:0006364,	K02875|1|4.55e-150|419|mmu:67115|large subunit ribosomal protein L14e	gi|13385472|ref|NP_080250.1|	###	60S ribosomal protein L14 [Mus musculus]
mmu-miR-378d	NM_001099631.1	7mer-m8	0	NA	-47.5	322	Sh2d5	GO:0014069,GO:0030054,GO:0045211,GO:0005515,	-	gi|153792029|ref|NP_001093101.1|	0	SH2 domain-containing protein 5 [Mus musculus]
mmu-miR-378d	NM_172578.2	7mer-1a	0	NA	-25.2	162	Mis18bp1	GO:0000778,GO:0003677,GO:0005515,GO:0051301,	-	gi|31044421|ref|NP_766166.2|	0	mis18-binding protein 1 [Mus musculus]
mmu-miR-378d	NM_001163502.1	8mer-1a	0	NA	-41.4	298	Elmsan1	GO:0005667,GO:0000118,GO:0044212,GO:0008134,GO:0003700,GO:0006357,	K11584|1|1.16e-13|81.6|adf:107331131|serine/threonine-protein phosphatase 2A regulatory subunit B'!K11829|2|3.45e-06|56.2|tru:101074846|REST corepressor 1	gi|254553410|ref|NP_001156973.1|	0	ELM2 and SANT domain-containing protein 1 [Mus musculus]

mmu-miR-378d	XM_006530496.3	6mer	0	NA	-28.7	163	Med131	-	K15164|1|0.0|4564|mmu:76199|mediator of RNA polymerase II transcription subunit 13	gi|568937551|ref|XP_006530559.1|	0	PREDICTED: mediator of RNA polymerase II transcription subunit 13-like isoform X1 [Mus musculus]
mmu-miR-378d	NM_177708.5	7mer-m8	0	NA	-48.6	304	Rtn4rl1	GO:0045121,GO:0046658,GO:0005737,GO:0009897,GO:0016021,GO:0070062,GO:0004860,GO:0004872,GO:0006469,GO:0019221,GO:0046426,GO:0031103,	K16660|1|0.0|919|mmu:237847|reticulon-4 receptor-like 1	gi|29244160|ref|NP_808376.1|	0	reticulon-4 receptor-like 1 precursor [Mus musculus]
mmu-miR-378d	NM_144868.3	6mer	0	NA	-30.9	164	Pcnx3	GO:0016021,	-	gi|569005509|ref|XP_006531676.1|	0	PREDICTED: pecanex-like protein 3 isoform X2 [Mus musculus]
mmu-miR-378d	NM_001038635.2	7mer-m8	0	NA	-26.7	164	Stk35	-	K08859|1|0.0|1106|mmu:67333|serine/threonine kinase 35 [EC:2.7.11.1]	gi|148696300|gb|EDL28247.1|	0	serine/threonine kinase 35 [Mus musculus]
mmu-miR-378d	XM_006530863.3	7mer-1a	0	NA	-46.9	317	Inpp4b	GO:0005794,GO:0016316,GO:0008289,GO:0034597,GO:0034594,GO:0051896,GO:0046822,GO:0046855,GO:0006874,GO:0046856,GO:0046850,GO:0045671,	K01109|1|0.0|1571|mmu:234515|inositol polyphosphate-4-phosphatase [EC:3.1.3.66]	gi|662236796|ref|NP_001284520.1|	0	type II inositol 3,4-bisphosphate 4-phosphatase isoform 1 [Mus musculus]
mmu-miR-378d	NM_007713.4	7mer-m8	0	NA	-44.5	287	Clk3	GO:0016607,GO:0045111,GO:0001669,GO:0016020,GO:0004674,GO:0004712,GO:0004713,GO:0005524,GO:0005515,GO:0043484,GO:0046777,GO:0018108,	K08823|1|0.0|575|pps:100976974|CDC-like kinase [EC:2.7.12.1]	gi|675711266|ref|XP_008961722.1|	0	PREDICTED: LOW QUALITY PROTEIN: dual specificity protein kinase CLK3 [Pan paniscus]
mmu-miR-378d	NM_009781.4	8mer-1a	0	NA	-61.7	450	Cacna1c	-	K04850|1|0.0|4370|rno:24239|voltage-dependent calcium channel L type alpha-1C	gi|755515972|ref|XP_011239477.1|	0	PREDICTED: voltage-dependent L-type calcium channel subunit alpha-1C isoform X6 [Mus musculus]

mmu-miR-378d	NM_026121.3	6mer	0	NA	-90.9	631	Bag4	GO:0005829,GO:0005886,GO:0005634,GO:0051087,GO:0031625,GO:0033138,GO:0030838,GO:0071364,GO:0097178,GO:0045785,GO:0090367,GO:0010763,GO:0072659,GO:0071356,GO:0042981,GO:0051496,GO:0051897,GO:2001145,GO:0051291,GO:1903215,	K09558|1|0.0|905|mmu:67384|BCL2-associated athanogene 4	gi|23273683|gb|AAH37239.1|	0	Bag4 protein, partial [Mus musculus]
mmu-miR-378d	NM_008368.4	7mer-m8	0	NA	-66.4	456	Il2rb	GO:0009897,GO:0016021,GO:0019976,GO:0004911,GO:0019221,GO:0030101,GO:0043066,	K05069|1|0.0|1104|mmu:16185|interleukin 2 receptor beta	gi|6680427|ref|NP_032394.1|	0	interleukin-2 receptor subunit beta precursor [Mus musculus]
mmu-miR-378d	XM_006497746.3	8mer-1a	0	NA	-69.7	481	Lmx1b	GO:0005634,GO:0008270,GO:0000977,GO:0005515,GO:0003700,GO:0045944,GO:0009953,GO:0071542,GO:0001701,	K09371|1|0.0|825|lve:103076536|LIM homeobox transcription factor 1	gi|568913075|ref|XP_006497809.1|	0	PREDICTED: LIM homeobox transcription factor 1-beta isoform X1 [Mus musculus]
mmu-miR-378d	NM_019750.3	7mer-m8	0	NA	-26.6	153	Nat6	-	-	-	-	-
mmu-miR-378d	NM_134437.3	8mer-1a	0	NA	-73.1	472	Il17rd	GO:0005887,GO:0005654,GO:0005794,GO:0046872,GO:0030368,	K05167|1|0.0|1518|mmu:171463|interleukin 17 receptor D	gi|568986950|ref|XP_006518730.1|	0	PREDICTED: interleukin-17 receptor D isoform X1 [Mus musculus]
mmu-miR-378d	NM_016852.2	7mer-m8	0	NA	-27.6	162	Wbp2	GO:0000790,GO:0005515,GO:0000979,GO:0001105,GO:0031490,GO:0045815,GO:0071391,GO:0045184,GO:0071442,GO:0045944,	-	gi|8394539|ref|NP_058548.1|	0	WW domain-binding protein 2 [Mus musculus]
mmu-miR-378d	NM_025648.3	7mer-m8	0	NA	-67.8	457	Farsa	GO:0009328,GO:0016020,GO:0005524,GO:0004826,GO:0000049,GO:0006432,GO:0051290,	K01889|1|0.0|1051|mmu:66590|phenylalanyl-tRNA synthetase alpha chain [EC:6.1.1.20]	gi|162138894|ref|NP_079924.2|	0	phenylalanine--tRNA ligase alpha subunit [Mus musculus]

mmu-miR-378d	NM_007511.2	7mer-1a	0	NA	-43.2	304	Atp7b	GO:0005887,GO:0032588,GO:0016323,GO:0005770,GO:0045177,GO:0005923,GO:0048471,GO:0005783,GO:0008270,GO:0004008,GO:0005507,GO:0005524,GO:0060003,GO:0007595,GO:0015677,GO:0006878,GO:0071287,GO:0015680,GO:0010043,GO:0006882,GO:0051591,GO:0051208,GO:0071280,GO:0007623,GO:1990637,	K17686|1|0.0|2949|mmu:11979|Cu+-exporting ATPase [EC:3.6.3.54]	gi|568953860|ref|XP_006509070.1|	0	PREDICTED: copper-transporting ATPase 2 isoform X1 [Mus musculus]
mmu-miR-378d	XM_006504740.3	7mer-m8	0	NA	-45.8	311	Iqce	GO:0005739,	-	gi|40254171|ref|NP_083109.2|	0	IQ domain-containing protein E [Mus musculus]
mmu-miR-378d	NM_016873.2	7mer-m8	0	NA	-25.3	155	Wisp2	GO:0005737,GO:0005615,GO:0009986,GO:0005886,GO:0005578,GO:0070062,GO:0008083,GO:0008201,GO:0005178,GO:0005520,GO:0060548,GO:0008285,GO:0001558,GO:0007155,GO:0007267,GO:0007165,	K06827|1|5.21e-60|194|lcm:102348379|connective tissue growth factor	gi|568916302|ref|XP_006499232.1|	###	PREDICTED: WNT1-inducible-signaling pathway protein 2 isoform X1 [Mus musculus]
mmu-miR-378d	XM_006510644.1	7mer-m8	0	NA	-44.5	303	Robo4	GO:0016021,GO:0009897,GO:0070062,GO:0004872,GO:0005515,GO:0001525,GO:0030336,GO:0030154,GO:0042981,	K06784|1|0.0|2003|mmu:74144|roundabout, axon guidance receptor 4	gi|823683831|ref|NP_001296319.1|	0	roundabout homolog 4 isoform 1 precursor [Mus musculus]
mmu-miR-378d	NM_001039530.3	7mer-m8	0	NA	-44.3	299	Parp14	-	K15261|1|0.0|3576|mmu:547253|poly [ADP-ribose] polymerase 10/14/15 [EC:2.4.2.30]	gi|568995875|ref|XP_006522456.1|	0	PREDICTED: poly [ADP-ribose] polymerase 14 isoform X3 [Mus musculus]
